# Application of Queuing Analytic Theory to Decrease Waiting Times in Emergency Department: Does it Make Sense?

**DOI:** 10.5812/atr.7177

**Published:** 2012-10-14

**Authors:** Mostafa Alavi-Moghaddam, Reza Forouzanfar, Shahram Alamdari, Ali Shahrami, Hamid Kariman, Afshin Amini, Shokooh Pourbabaee, Armin Shirvani

**Affiliations:** 1 Emergency Department, Imam Hossein Hospital, Shahid Beheshti University of Medical Sciences, Tehran, IR Iran; 2 Office of Health And Medical Education Hospital Standards, Ministry of Health, Tehran, IR Iran

**Keywords:** Emergency Department, Operational Research, Queuing, Quality Improvement

## Abstract

**Background:**

Patients who receive care in an emergency department (ED), are usually unattended while waiting in queues.

**Objectives:**

This study was done to determine, whether the application of queuing theory analysis might shorten the waiting times of patients admitted to emergency wards.

**Patients and Methods:**

This was an operational study to use queuing theory analysis in the ED. In the first phase, a field study was conducted to delineate the performance of the ED and enter the data obtained into simulator software. In the second phase, "ARENA" software was used for modeling, analysis, creating a simulation and improving the movement of patients in the ED. Validity of the model was confirmed through comparison of the results with the real data using the same instrument. The third phase of the study concerned modeling in order to assess the effect of various operational strategies, on the queue waiting time of patients who were receiving care in the ED.

**Results:**

In the first phase, it was shown that 47.7% of the 3000 patient records were cases referred for trauma treatment, and the remaining 52.3% were referred for non-trauma services. A total of 56% of the cases were male and 44% female. Maximum input was 4.5 patients per hour and the minimum input was 0.5 per hour. The average length of stay for patients in the trauma section was three hours, while for the non-trauma section it was four hours. In the second phase, modeling was tested with common scenarios. In the third phase, the scenario with the addition of one or more senior emergency resident(s) on each shift resulted in a decreased length of stay from 4 to 3.75 hours. Moreover, the addition of one bed to the Intensive Care Unit (ICU) and/or Critical Care Unit (CCU) in the study hospital, reduced the occupancy rate of the nursing service from 76% to 67%. By adding another clerk to take electrocardiograms (ECG) in the ED, the average time from a request to performing the procedure is reduced from 26 to 18 minutes. Furthermore, the addition of 50% more staff to the laboratory and specialist consultations led to a 90 minute reduction in the length of stay. It was also shown that earlier consultations had no effect on the length of stay.

**Conclusions:**

Application of queuing theory analysis can improve movement and reduce the waiting times of patients in bottlenecks within the ED throughput.

## 1. Background

With increasing demand and shortage of resources, waiting time is an inevitable problem in all clinical fields. However, it is particularly important for emergency departments (ED), where the optimum use of limited resources is critical. Optimizing patient turnover, decisions to eliminate bottlenecks and blocks in patient flow and service delivery in key sectors will potentiate the system to reduce costs and improve quality of care. The ED is one of the most overcrowded units in the inpatient service delivery system. Delays in services in the ED may have unpleasant consequences for patients. Under such circumstances, the important point for hospitals is the development of a scientific methodology to improve their clinical approach, preparing the most cost effective level of care, in the appropriate time, with the most efficient use of limited resources. Applying operations research (OR) (1) in health care planning, is a well-known method for the optimal use of resources and reduction in waiting times. These researches provide useful tools in decision making, using mathematical models and simulation of operations in existing capacities, such as the ED. The basis for such analysis and simulation approaches is the analysis of past events, their statistical simulation and extrapolation of possible future events with an acceptable percentage of error. These kinds of studies are used when the systems are so complex that their behavior cannot be easily predicted, in order to formulate interventions based on those arrangements. The more complicated the system, the greater the number of possible input elements and relationships are created. One type of research is called, 'queuing theory analysis' (2). It is used in areas where the sequence of events and waiting times are very important. In fact, queuing theory is a mathematical method for analyzing expected times, on the basis of which planning to provide capacity and service can be made. Waiting queues usually appear from the random entry of patients to the ED. It is possible to balance the cost of the patients’ time wasted waiting and the costs of the services provided by the application of this method. This theory, with the use of mathematical models and statistical distributions, can help managers to improve their system functions by analyzing delays and waiting times during the processes.

### 1.1. Modeling of Emergency Department Using Computerized Software Simulation

Emergency departments contain a heterogeneous set of queues and service points. Generally, there are two different methods including; queuing analysis and discrete event modeling approach, for the quantitative assessment of these systems. During the past thirty years, many researches have been conducted in the field analysis of ED using discrete event simulation (3-7). In a study by Rossetti et al. the effects of virtual changes in the working schedules of emergency physicians on patients and emergency resources were reviewed (8).In a study by Loyd and Bair in 2006, two triage “EDSIM” and ‘Acuity Ratio Triage (ART)’ were compared and this showed that triage on the basis of the acuity of illness, ART, reduced the imaging time for a group of patients (6). There have been few studies on field analysis using line and computer reconstruction for upgrading emergency functioning.

## 2. Objectives

There has been no study conducted using queuing analysis and computer simulation in ED’s in IR Iran; therefore, this study was designed to use these techniques with the purpose of improving system functioning by testing bottleneck scenarios.

## 3. Patients and Methods

**Figure 1.**
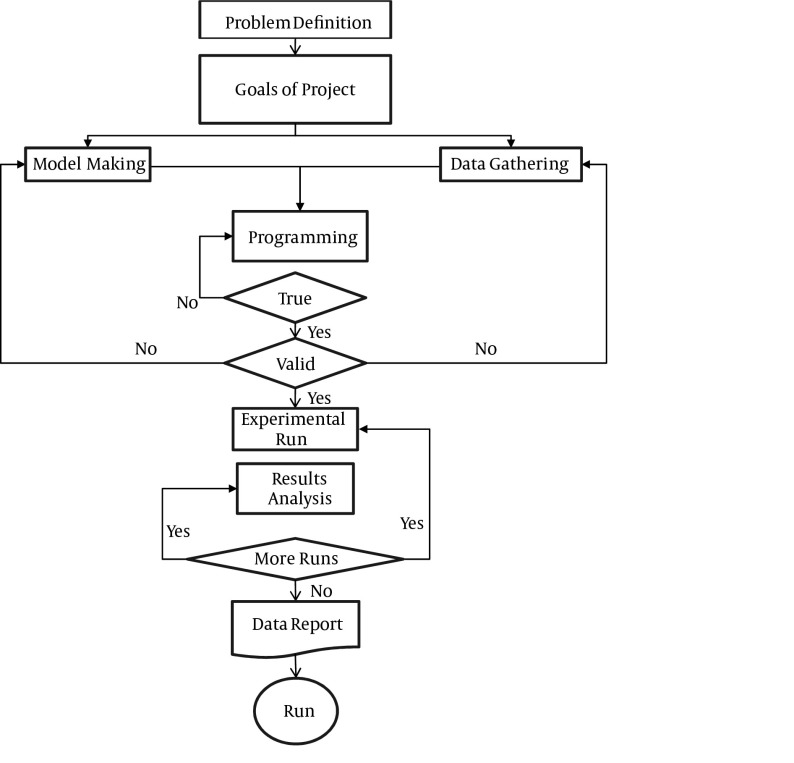
General Steps of Queuing Model Analysis

General information about the study environment: The Imam Hosein Hospital was selected for this study. It is the main hospital of the Shahid Beheshti University of Medical Sciences, located in the east of Tehran, Iran and it has an ED with an annual input of 50000 patients. Patients enter the triage room of the ED either by self-referral, outpatient referral, or through emergency medical services (EMS). In cases where there is any instability in; vital signs, level of consciousness or cardiac function; those patients are taken directly to the resuscitation room. Otherwise, a trained nurse based on an installed ESI - 4 triage system guides the patients, in 24 hours a day. Patients, in this system are divided into five priority groups. Groups 1, 2 and 3 are admitted to the ED, and groups 4 and 5 are referred to a first year emergency medicine resident in an adjacent room. The chief complaints of the patients are recorded in a triage sheet (Figure 1).

### 3.1. Study Design

This study was conducted in three phases:

I) Data collection and identification of critical factors

II) Making a model 

III) Testing scenarios

### 3.2. First Phase (Field Study)

In this cohort study, questionnaires were completed by trained nurses who are present 24 hours a day, seven days a week, during two consecutive months. Data included demographic items and all service times. Accuracy of the data was confirmed by comparison of the hospital informatics center data for the same patients from their authenticity and manual methods. Data was entered into an excel data sheet.

### 3.3. Second Phase: Modeling the Flow of Emergency Patients 

The flow of patients in the ED is very complex (7), so that even patients with similar complaints may pass through different routes. Emergency patients usually pass through three separate routes:

### 3.3.1. Input

1) Interval between triage logging and chart recording 

2) Interval between chart recording and the first visit of the Emergency Medical Resident

### 3.3.2. Throughput

3) Interval between the emergency medical resident’s first visit and checking the orders by the emergency nurse

4) Interval between the first ECG order and the first ECG

5) Interval between the first lab data order and the first samples obtained

6) Interval between the first request and performing the ultrasound

7) Interval between the first CT scan order and first CT scan

8) Interval between the first request for an X - ray and its performance

9) Interval between the first request for a specialist consultation and its performance

### 3.3.3. Output

10) Interval between the first order for admission to the CCU and admission

11) Interval between the first order for admission to the ICU and admission

12) Interval between the first order to send the patient to the operating room and its performance

13) Interval between the first order to transfer the patient to other wards and its performance

14) Interval between the first order of discharge from the emergency and its performance 

Time of death and patient personal satisfaction were recorded as well

### 3.4. Software Modeling

ARENA v 13.5 software, (Rochwell, USA) was used as the common model for the flow of patients in the ED. The following were assumed; total number of beds, without extra-beds, in general emergency was assessed as 24 beds. Number of nursing staff for every shift was eight. The numbers for trauma emergency were 14 beds and four nurses. The number of residents per shift was six, including; three 1st year residents, two 2nd years and one 3rd year. The capacity of the radiology and laboratory services was assumed to be constant. It was also, assumed that one ECG technician was present on each shift.

### 3.5. Data Analysis

Waiting time for each patient was recorded in Microsoft Office Excel 2007, and analyzed in SPSS 20 software, (IBM). Then, the intervals obtained in the first phase of the study were entered into the software. The maximum and minimum inputs of each day including holidays (from 12 a. m of Thursday to the 12 a. m of Friday), both day and night were calculated for a normal week.

**Figure 2.**
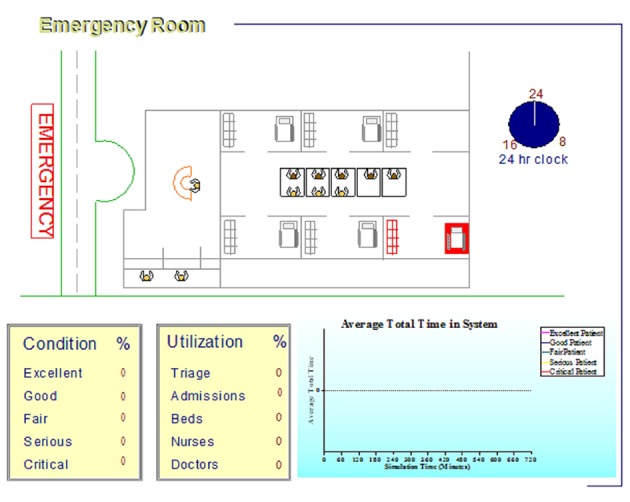
Simulated Computer Model of Emergency Department

In phase III of the study, the scenarios were tested by Arena software, data visualization and animation by Simul8 v 9.0 software. Changes in any of the bottlenecks, resulting expenses and the impact on patients waiting times were all examined (Figure 2).

## 4. Results

### 4.1. Results of the First Phase (Field Study)

By the completion of the first phase of the study, data from 3000 patients had been recorded. In total, 40 chief complaints were the main causes of patients’ referral to the ED. They were categorized into eight specific groups for the ease of the study; general, cardiovascular, respiratory, neurological, psychiatric, traumatic, toxicity and surgical problems. Direct admission of psychiatric patients and obstetrics cases to the related wards, is the reason for the absence of data in these fields. The time intervals obtained for each group of complaints is shown in Table 1.

**Table 1:** Demographic Characteristics and Number of Patients Entering the Emergency Ward

	Variant
**Age, y, Median (Range) **	39 (0 - 90)
**Age, y, Mean ± SD**	42 ± 22
**Gender, No. (%)**	
Male	2680 (56.0)
Female	1320 (44.0)
**Shift, No. (%)**	
Day	1470 (50.9)
Night	1420 (49.1)
**Entrance Type, No. (%)**	
Ambulatory	250 (8.6)
Emergency medical services	2670 (91.4)
**Triage, No. (%)**	
1	40 (1.4)
2	40 (1.4)
3	2470 (84.3)
4	350 (11.9)
5	30 (1.0)
**Complaint, No. (%)**	
Cardiac	240 (8.4)
General	620 (21.6)
Respiratory	170 (5.9)
Trauma	1370 (47.7)
Toxicology	40 (1.4)
Psychology	0 (.0)
Surgery	60 (2.1)
Neurological	370 (12.9)

**Figure 3.**
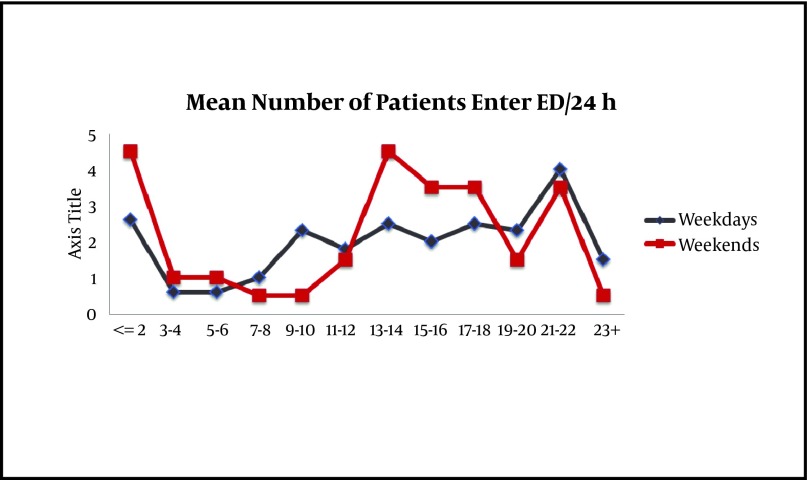
Separate Entrance for Patients: Normal and Holidays, 24 hours

The highest number of entries was 125 patients and the lowest input was 37 patients per day. The maximum patient input was 4.5, and the minimum was 0.5 persons per hour, between 2:00 to 8:00 PM, and 7:00 to 8:00 AM, respectively. Circadian distribution of patients (from 7:30 AM to 7:30 PM) was almost the same. The average length of time that patients stayed in the ED was four hours. Mean time of leaving the ER was 28 minutes. In general, the minimum interval was for the first visit with four minutes, and the maximum time was for an ICU request with 1028 minutes (Figure 3).

### 4.1.1. Input

The average interval for recording was 11 minutes, but this ranged from zero to 77 minutes. Half of the patients were recorded within eight minutes. In addition, according to Table 2, approximately 13% of the patients were allocated to groups 4 and 5 triage class, and were in fact re-triaged.

**Table 2:** General Service Intervals

	No. (%)	Mean ± SD	Minimum	25^th^	Median	75^th^	Maximum
**Interval registration**	2890 (96.3)	11 ± 10	0	5	8	13	77
**Interval order**	2880 (96)	4 ± 8	-57	0	5	5	55
**Interval electro cardio gram**	1090 (36.3)	26 ± 46	-4	5	10	25	295
**Interval Lab**	1540 (51.3)	87 ± 108	0	10	55	120	560
**Interval sonography**	610 (20.3)	94 ± 85	-5	35	65	130	405
**Interval computerized scan**	860 (28.7)	116 ± 93	5	60	80	145	480
**Interval X-ray**	1750 (58.3)	74 ± 71	0	30	60	105	470
**Interval consultation**	530 (17.7)	166 ± 134	5	70	130	230	690
**Interval CCU**	0 (0)	0 ± 0	0	0	0	0	0
**Interval intensive care unit**	50 (1.7)	1028 ± 616	75	735	1380	1450	1500
**Interval operating room**	10 (0.3)	680 ± 0	680	680	680	680	680
**Interval other**	120 (4)	330 ± 386	15	45	173	487	1185
**Interval pexit, h**	1700 (56.7)	4 ± 3	0	3	4	6	19
**Interval discharge, h**	2270 (75.7)	4 ± 3	0	2	3	5	25
**Interval disexit**	1530 (51)	28 ± 36	0	15	20	30	412
**Interval death, h**	80 (2.7)	93 ± 118	1	4	26	184	311
**Interval own satisfaction, h**	320 (10.7)	5 ± 4	0	2	4	7	19

### 4.1.2. Throughput

The longest interval in this group was for a consultation with 166 minutes, and the minimum for ordering with four minutes. The mean interval for the first visit was four minutes, with a range of minus 57 minutes to 55 minutes. The negative value represents a first visit that was made before the service record, because of the instability of the patient’s condition or due to a delay in preparation of the chart. The average time interval for an ECG request to its performance was 26 minutes, ranging from minus four to 295 minutes. The average interval between the request of a lab data and its performance was 87 minutes, ranging from zero to 560 minutes with a median of 55 minutes. Other intervals are given in Table 2.

### 4.1.3. Output

The longest waiting time was for ICU with 1028 minutes and the minimum for a transfer to other wards with 330 minutes.

### 4.2. Third Phase: Scenario Testing

In this step, the capacity of some fields such as CT scans and the laboratory was not changeable, and so they were considered to be constant, while the number of staff or residents would be changeable. The effects of the eight scenarios were tested on waiting times.

### 4.2.1. Input

1) Scenario: Adding one first year resident for each 12-hour shift. The average interval for the first order, which was the duty of a first year resident, was four minutes that resulted in an average four hour in-patient stay. The addition of another first year resident had no effect on the interval.

### 4.2.2. Throughput

2) Scenario: The addition of one third year resident for each 12 hour shift resulted in no significant change (P =0.07). The mean length of stay was reduced from 4 hours to 3.75 hours in all categories (P = 0.06).

3) Scenario: Adding one nurse to take ECG’s for each 12 hour shift resulted in a reduction of waiting time from 26 to 18 minutes. This is statistically significant P < 0.05.

4) Scenario: Increasing the laboratory capacity by 100% reduced the average stay by about 45 minutes (almost half of a lab data interval). It is statistically significant (P = 0.04).

5) Scenario: Increasing consultation capacity by 50% decreased the average patient stay in the emergency department by about 45 minutes, (P = 0.04).

6) Scenario: A simultaneous 50% increase in the capacity of both consultation and laboratory capacities, decreased the average time of a patient’s stay as much as 90 minutes and the average length of stay for all groups to three hours.

### 4.2.3. Output

7) Scenario: Reducing waiting times for patients needing the CCU and ICU. This change should be calculated by the discrete event calculation method. Moreover, by the addition of an emergency ICU/CCU bed, nursing service occupation was reduced from 78% to 67%.

8) Scenario: Discharge capacity enhancement. In general, the average occupancy rate of discharge capacity was 97.6% which approaches 114% on peak working days. By increasing the capacity of this unit 50%, the overall occupancy and peak of activity was halved. However, the overall average of patients’ stays showed no significant reduction (average 4 hours versus 3.96, (P = 0.07). The average leaving physically time decreased from 28 minutes to approximately 26 minutes.

## 5. Discussion

This study showed that the application of queuing theory analysis can significantly improve the flow of patients and reduce waiting times in bottlenecks in throughput in the ED. In other words, it can reduce waiting times in the queue for any of the emergency care procedures in the ED.

1) Triage: Approximately 13% of patients were re-triaged, (patients in the 4th and 5th triage level). This was due to the specific diseases or conditions of the referring patients, on the one hand, or entering the ED to receive care directly, on the other hand.

A) Installation of a fast track system is recommended for this group of patients as a separate section within the main emergency ward. Pinney et.al. found that a fast track may be useful in improving overall patients’ stay time (8).

B) The average chart record interval was 11 minutes, which is not appropriate for a critically ill patient. Using a computerized system in the triage room, or using hospital information system (HIS) compatible, bar coded wristbands, during the patient’s movement, would help to allocate resources to the right places, at the right times and inadequate amounts to the appropriate patient.

2) Patients hospital stay: The average patients’ stay in the ED was four hours.

Increasing either the laboratory or consultation capacity scenarios resulted in a similar reduction in the average patients’ length of stay, while a simultaneous increase in both services led to twice the average reduction. In the modeling scheme this study consultation was planned after obtaining the lab data, in other words, it was considered as a series in a circuit. So, having the consultations before the lab results not only will not change the staying time, but also, can theoretically increase the number of consultations and thus the timing. Although mean turnover was decreased to three hours in the study, however, it should be noted that many cases of trauma, or those with complaints of chest pain, or suspected ischemic heart disease, should have a minimum observation time in the emergency room, based on emergency guidelines (9).

A) Using real time line analysis software allows emergency managers to find the causes of ED overcrowding, and help them to improve it. Moreover, the hospital could be better managed without an inappropriate increase in staff numbers, but only by changing the position of staff from the ED to the wards or vice versa.

B) The addition of a clerk responsible for taking an ECG strip: Service time was reduced from 26 to 18 minutes, but it was still undesirable. According to the standard timing for patients with chest pain, it seems that the number of ECG machines is too low. The door to the ECG standard time interval is below five minutes (6).

C) Moreover, the time interval between obtaining the strip and its evaluation by a physician was not recorded well and this also emphasizes the importance of a trial to reduce this time.

3) Holidays: Considering the peak of work during vacations and the inadequate effect of junior residents on the work load in the ED, it is recommended that at least during these times, a senior resident should be added to the system. Due to the relationship between the first visit interval and patient satisfaction, a study on this item seems to be warranted.

4) We recommend an independent radiology department, especially CT scan for the ED, particularly on busy days.

### 5.1. Output

1- In this study, the average access time to the ICU was 1028 minutes. The addition of one bed reduced emergency nurses work load in each from 78% to 67%. This has not been referred to in similar studies (8).

2- Increasing the discharge capacity by 50% led to a 50% decline in occupancy capacity. However, although it could reduce working pressure, it had no significant effect on the average patients’ length of stay (about 2 minutes). The mean interval of physical discharge decreased from 28 minutes to about 26 minutes, indicating that despite the increased capacity it has not caused any significant change (P > 0.05). So other factors may also be effective at this point. One of the causes of overcrowding in the ED are those patients who are going to be discharged, but they are waiting for a final paraclinical procedure, such as a CT scan or radiography for the final decision. Formation of a section in emergency rooms with limited monitoring facilities and staff with the name, ‘Ready for Discharge Unit’ may be a helpful way to control the load (10-12). It can be part of the discharge area. Of course it would be better to install it after a period of modeling. In the 2012 guidelines for reducing overcrowding in ED’s, a discharge lounge to increase the speed of discharge is recommended (www.collemergencymed.ac.uk).

3- Clearance interval: The average physical withdrawal of the emergency beds was 28 minutes, but more than half of the patients occupied the bed for more than 20 minutes after discharge. Discharge areas may be useful for this purpose.

C) The emergency secretary desk may also be placed in this location.
